# Protein-protein interaction extraction with feature selection by evaluating contribution levels of groups consisting of related features

**DOI:** 10.1186/s12859-016-1100-z

**Published:** 2016-07-25

**Authors:** Thi Thanh Thuy Phan, Takenao Ohkawa

**Affiliations:** Department of Information Science, Graduate School of System Informatics, Kobe University, 1-1, Rokkodai, Nada, Kobe, 657-8501 Japan

**Keywords:** Biomedical text mining, Information extraction, *k*-nearest neighbors, Protein protein interaction

## Abstract

**Background:**

Protein-protein interaction (PPI) extraction from published scientific articles is one key issue in biological research due to its importance in grasping biological processes. Despite considerable advances of recent research in automatic PPI extraction from articles, demand remains to enhance the performance of the existing methods.

**Results:**

Our feature-based method incorporates the strength of many kinds of diverse features, such as lexical and word context features derived from sentences, syntactic features derived from parse trees, and features using existing patterns to extract PPIs automatically from articles. Among these abundant features, we assemble the related features into four groups and define the contribution level (CL) for each group, which consists of related features. Our method consists of two steps. First, we divide the training set into subsets based on the structure of the sentence and the existence of significant keywords (*SKs*) and apply the sentence patterns given in advance to each subset. Second, we automatically perform feature selection based on the CL values of the four groups that consist of related features and the *k*-nearest neighbor algorithm (*k*-NN) through three approaches: (1) focusing on the group with the best contribution level (BEST1G); (2) unoptimized combination of three groups with the best contribution levels (U3G); (3) optimized combination of two groups with the best contribution levels (O2G).

**Conclusions:**

Our method outperforms other state-of-the-art PPI extraction systems in terms of *F*-score on the HPRD50 corpus and achieves promising results that are comparable with these PPI extraction systems on other corpora. Further, our method always obtains the best *F*-score on all the corpora than when using *k*-NN only without exploiting the CLs of the groups of related features.

## Background

Identifying protein-protein interactions (PPIs) is necessary for constructing protein interaction networks and increases the understanding of both the functional role of individual proteins and the underlying biological processes. Although numerous PPIs have been manually curated by biomedical curators into databases, such as DIP, BIND, MINT, IntAct, and HPRD, many valuable PPIs remain available in research articles. Since the manual curation of relevant PPIs from millions of research articles is too time-consuming, methods of automatic PPI extraction from articles are necessary.

Many automatic PPI extraction methods from articles have been developed. The first, simplest kind of method, co-occurrence, which classifies two proteins as interacting if they exist in the same sentence or in the same abstract, yields high recall but low precision. Conversely, the second kind of method, pattern- or rule-based, which often utilizes handcrafted patterns or rules, achieves high precision but low recall. The third kind of method, machine learning-based, can be divided into two types: feature-based and kernel-based.

Feature-based methods represent an instance, which is a protein pair that consists of two protein names in a sentence, by many features, e.g., lexical, word context, and syntactic features derived from the sentence or its syntactic structure. Besides lexical and syntactic features, Liu et al. [1] exploited various features from dependency information, including predicate features involved in dependency graphs. Landeghem et al. [2] proposed rich feature vectors containing semantic information from dependency graphs and lexical information from sentences. They also first applied automatic feature selection techniques and showed that these techniques can enhance the generalization performance in PPI extraction and make the models faster and more cost-effective.

Recently, various kernel-based methods have been proposed that provide kernel functions that measure the similarity between any pair of instances represented by structural representations, such as constituent parse trees or dependency graphs. These kernel functions differ from each other in their type of input representation and how they compute similarity functions. Airola et al. [3] proposed a graph kernel-based method in which a sentence is represented by a combination of a dependency graph and another graph showing the linear order of words and considered all the possible paths connecting two entities in the dependency graph. Miwa et al. [4] proposed a method that combines kernels based on several syntactic parsers to retrieve the widest possible range of important information from a given sentence. Their method, which combines a bag-of-words kernel, a subset tree kernel, and a graph kernel, assigns the same weight to each individual kernel. Qian et al. [5] proposed a tree kernel-based method by exploiting both constituent parse trees and dependency graphs and further refined the tree representation from a constituent parse tree by utilizing the shortest dependency path between two proteins in a dependency graph.

Feature-based methods are considered more appropriate to practical applications than kernel-based methods due to the computation complexity of sophisticated kernels [1]. Moreover, feature-based methods can be improved by applying feature selection to enhance the generalization performance and attain faster and more cost-effective models [2]. The latest study of Tikk et al. [6] analyzed and compared the performances of diverse kinds of kernels. They found that different kernels using the same input representation perform similarly on a large number of protein pairs, which are identified as misclassified by most state-of-the-art kernel-based methods. Based on their convincing experimental results, to improve the PPI extraction performance, they argued that we should concentrate on finding and choosing informative features suitably rather than devising novel similarity functions encoded in kernels.

In this paper we propose a novel feature-based method to extract PPIs from articles. We exploit various features, including lexical features and word context features obtained directly from sentences, syntactic features obtained from parse trees, and features using existing patterns. We arrange the related features into four groups. For instance, we assemble two related features, which represent the positions of two protein names that constitute an instance in the sentence containing them, into one group. We also define the contribution level (CL) of each group, which consists of related features. Our method differs from existing methods in two ways. First, based on the structure of the sentence and the presence of significant keywords (*SKs*), we divide the training set into subsets and apply the sentence patterns provided beforehand to each one. Second, after computing the CL values of the four groups that consist of related features, we automatically implement feature selection based on their CLs and the *k*-nearest neighbor algorithm (*k*-NN) through three approaches: (1) focusing on the group with the best contribution level (BEST1G); (2) unoptimized combination of three groups with the best contribution levels (U3G); (3) optimized combination of two groups with the best contribution levels (O2G). To the best of our knowledge, this is the first method that automatically selects the most appropriate features for each group consisting of related features by combining their CLs with *k*-NN.

**Table 1:** Lexical features obtained directly from sentences

Features	Definitions/Remarks	Values	Examples
Keyword	Words indicating relationship between two proteins.	One of the 180 kinds of words obtained by stemming 642 kinds of words such as *‘bind’*, *‘link’*, *‘stimulate’*, *‘interact’*,*‘induce’*, *‘regulate’*, *‘mediate’*,*‘inhibit’*, etc., which often exist in sentences containing PPIs.	In sentence IEPA.d0.s0 (Fig. 1), feature *keyword*’s value is *‘stimulate’*.
Negative word	Check if one such negative word as *‘not’*, *‘incapable’*, and *‘unable’* appears between *keyword* and one of the two protein names or between two protein names.	‘true’ or ‘false’	In sentence HPRD50.d21.s1 of HPRD50 corpus, *“In contrast to OX1R, the potency of direct activation of CB1 was not affected by co-expression with OX1R,”* feature value is ‘true’.
Conjunctive word	Check if one of the following words indicating a conjunctive relation appears: *‘although’*, *‘though’*, *‘because’*, *‘as’*, *‘therefore’*, *‘hence’*, *‘since’*, *‘so’*, *‘where’*, *‘when’*, *‘what’*, *‘why’*, *‘how’*, *‘wherein’*, *‘whereas’*, and *‘whereby’*.	‘true’ or ‘false’	In sentence HPRD50.d21.s1 above, feature value is ‘false’.
‘Which’	Check if ‘which’ appears. Although ‘which’ also shows conjunctive relations, because ‘which’ appears more often than the conjunctive words listed above, we differentiate it from the above features.	‘true’ or ‘false’	In sentence LLL.d13.s0 of LLL corpus, *“Production of sigmaK about 1h earlier than normal does affect Spo0A, which when phosphorylated is an activator of sigE transcription,”* feature value is ‘true’.
‘But’	Check if ‘but’ appears. Although ‘but’ also appears as frequently as ‘which’ to represent conjunctive relations, ‘but’ implies negation of context.	‘true’ or ‘false’	In sentence AIMed.d55.s485 of AIMed corpus, *“LEC also induced calcium mobilization, but marginal chemotaxis via CCR5,”* feature value is ‘true’.
Words indicating condition or presumption	Check if ‘if’ or ‘whether’ appears between *keyword* and one of the two protein names or between two protein names.	‘true’ or ‘false’	In sentence IEPA.d0.s0 (Fig. 1), feature value is ‘false’.
Preposition of keyword	Preposition following *keyword* providing that the distance between it and the *keyword* is within 3. If there are many prepositions, the preposition closer to the *keyword* is utilized.	One of the prepositions	In sentence AIMed.d55.s487 of AIMed corpus, *“The binding of LEC to CCR8 was much less significant,”* feature value is ‘of’.
Second keyword	Only one of seven words, *‘bind’*, *‘interact’*, *‘stimulate’*, *‘associate’*, *‘regulate’*, *‘induce’*, and *‘known’*, is not chosen as a *keyword*, check if that word appears between two protein names. These seven words can be considered especially significant in PPI classification compared with other *keywords*. This feature prevents these words from being overlooked as *keywords*.	‘true’ or ‘false’ for each of these seven words (If one of these seven words appears in the sentence and is not chosen as a *keyword*, feature value for it is ‘true’).	In sentence IEPA.d0.s0 (Fig. 1), because ‘stimulate’ was already chosen as a *keyword* and the sentence does not contain the other six words, feature value of the second keyword (*‘stimulate’*) is ‘false’ and feature value of the other six words is also ‘false’.

Lexical features extracted from sentences

## Outline of features of protein pairs in sentences used in extraction of protein-protein interaction information

The automatic extraction of PPI information from articles is regarded as a binary classification in which instances (protein pairs) that include PPI and instances that do not include PPI are classified as positive and negative instances. In machine learning approaches, based on labeled training data, extracted features, which are the characteristics of sentences containing protein pairs, are utilized to discriminate positive from negative instances. Then a model is learned from the training data, and each instance is classified as positive or negative.

**Fig. 1:**
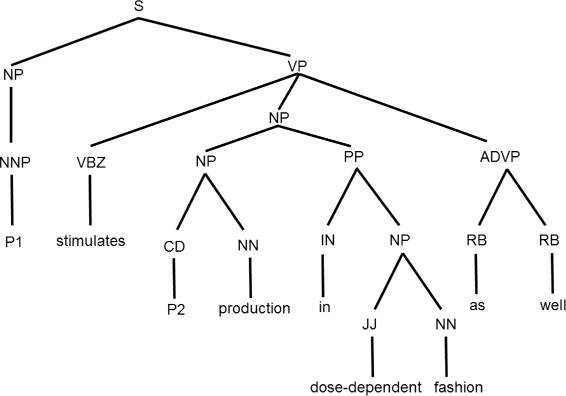
Example of a constituent parse tree. Constituent parse tree for sentence, *“Oxytocin stimulates IP3 production in dose-dependent fashion as well,”* from sentence IEPA.d0.s0 of IEPA corpus (first protein *P*1 is Oxytocin and second protein *P*2 is IP3)

Our features are categorized into four types: lexical features obtained directly from the sentence, word context features obtained directly from the sentence, syntactic features obtained from the parse tree, and features that use the existing patterns. In the following tables, *P*1, *P*2, and *K* denote the protein name that appears first, the protein name that appears later, and the *keyword* in a sentence, respectively.

- Lexical features obtained directly from sentences: These features are outlined in Table 1.
- Word context features obtained directly from sentences: These features are outlined in Table 2.

**Table 2:** Word context features obtained directly from sentences

Features	Definitions/Remarks	Values	Examples
Distance_KP1	The distance defined by number of words appearing between *keyword* *K* and protein name *P*1 in the sentence.	Integer value	In sentence LLL.d33.s1 of LLL corpus, *“GerE binds to a site on one of these promoters, cotX, that overlaps its -35 region,”* *keyword* is ‘bind’ and Distance_KP1 is 0.
Distance_KP2	The distance between *keyword* *K* and *P*2 in the sentence.	Integer value	In sentence LLL.d33.s1 above, Distance_KP2 is 8.
Distance_P1P2	The distance between two protein names in the sentence.	Integer value	In sentence LLL.d33.s1 above, Distance_P1P2 is 9.
Position_P1	The value adding word distance between protein name *P*1 and beginning of the sentence to one.	Integer value	In sentence LLL.d33.s1 above, Position_P1 is 1.
Position_P2	The value adding word distance between protein name *P*2 and beginning of the sentence to one.	Integer value	In sentence LLL.d33.s1 above, Position_P2 is 11.
Position of keyword	The word order of *keyword* *K* and protein pair *P*1 and *P*2. ‘Infix’: order of words is [ *P*1-*K*- *P*2]), ‘prefix’: order of words is [*K*- *P*1- *P*2]), or ‘postfix’: order of words is [ *P*1- *P*2-*K*]).	‘Infix’, ‘prefix’, or ‘postfix’	In sentence LLL.d33.s1 above, feature value is ‘infix’.
Comma between keyword and protein pair	Because topic of the sentence frequently changes before and after commas, we utilize the information if there is a comma between protein pair and *keyword*. ‘ *x*_1_ *x*_2_’: *x*_1_ is ‘t’ if a comma exists between *A* and *B*, and *x*_2_ is ‘t’ if a comma exists between *B* and *C*, otherwise *x*_1_ or *x*_2_ is ‘f’, where A, B, and C represent a *keyword* and two protein names in order of their appearance in the sentence.	‘tt’, ‘ff’, ‘tf’, or ‘ft’	In sentence LLL.d33.s1 above, feature value is ‘ft’.
Multiple occurrences of keywords	Check whether there is more than one *keyword* in a sentence.	‘true’ or ‘false’	In sentence LLL.d33.s1 above, feature value is ‘false’.
Parallel expression of a protein pair	Check whether the two protein names of the protein pair are contiguous in the word order of the sentence containing them (they are also considered contiguous even if *‘-’*, *‘/’*, *‘and’*, *‘or’*, *‘(’* appears between them). If two protein names are described in parallel in a sentence, an interaction between them is unlikely.	‘true’ or ‘false’	In sentence LLL.d30.s0, *“In vitro, both sigma(A) and sigma(X) holoenzymes recognize promoter elements within the sigX-ypuN control region,”* feature values of protein pairs (sigma(A), sigma(X)) and (sigX, ypuN) are ‘true’ (only PPIs are in the remaining protein pairs).

Word context features extracted from sentences. *P*1, *P*2, and *K* denote the protein name appearing first, the protein name appearing later, and the *keyword* in a sentence, respectively. ‘t’ and ‘f’ are abbreviations of ‘true’ and ‘false’
- Syntactic features obtained from parse trees: All sentences are transformed into representations called constituent parse trees, which can capture the syntactic structures of sentences. The features obtained from the constituent parse tree are outlined in Table 3.

**Table 3:** Syntactic features obtained from parse trees

Features	Definitions/Remarks	Values	Examples
Height_P1	The height of first protein name *P*1 of instance at constituent parse tree. This height differs from features Distance_KP1, Distance_KP2, and Distance_P1P2, i.e., distances between protein pair and *keyword* described in Table 2.	Integer value	In Fig. 1, Height_P1 is 2.
Height_P2	The height of second protein name *P*2 of instance at constituent parse tree. This height differs from features Distance_KP1, Distance_KP2, and Distance_P1P2.	Integer value	In Fig. 1, Height_P2 is 4.
Height_K	The height of *keyword* *K* at constituent parse tree. This height differs from features Distance_KP1, Distance_KP2, and Distance_P1P2.	Integer value	In Fig. 1, Height_K is 2.
POS_P1	We take into account the part-of-speech information of path from root at constituent parse tree of the two protein names constituting instance and *keyword*. It is possible to represent syntax structure and train classifiers to learn the pseudo grammar structure by this information. POS_P1 denotes part-of-speech information of path from root of leaf representing first protein *P*1 of instance.	The list of part-of-speech information of path from root at constituent parse tree.	In Fig. 1, POS_P1 is ‘NP, NNP’.
POS_P2	POS_P2 denotes part-of-speech information of path from root of leaf representing second protein *P*2 of instance.	The list of part-of-speech information of path from root at constituent parse tree.	In Fig. 1, POS_P2 is ‘VP, NP, NP, CD’.
POS_K	POS_K denotes part-of-speech information of path from root of leaf representing *keywork* *K*.	The list of part-of-speech information of path from root at constituent parse tree.	In Fig. 1, POS_K is ‘VP, VBZ’.

All sentences were transformed into representations called constituent parse trees, output from the Stanford parser [7]. Syntactic features were extracted from constituent parse trees. *P*1, *P*2, and *K* denote the protein name appearing first, the protein name appearing later, and the *keyword* in a sentence, respectively
  An example of a constituent parse tree output from the Stanford parser [7] is shown in Fig. 1.
- Features using existing patterns: We prepared thirteen syntax patterns related to the presence or absence of PPI in Table 4 based on the syntax patterns that were proposed by Plake et al. [8]. iNoun and iVerb, which represent the sets of nouns and verbs related to interaction, are improved from the original ones used by Plake et al. [8]. The wildcard ‘*’ represents any word or words in a pattern. The number of words substituted by a wildcard in a pattern is limited to five. If an instance (a protein pair (*P*1,*P*2)) matches (or does not match) one of these patterns, the feature value is set as ‘true’ (or ‘false’).

**Table 4:** Set of PPI syntax patterns

No.	PPI-Pattern
Pattern 1	**P1** ^∗^ **iVerb** ^∗^ **P2**
Pattern 2	**P1** ^∗^ **iVerb** ^∗^ by ^∗^ **P2**
Pattern 3	**iVerb** of ^∗^ **P1** ^∗^ by ^∗^ **P2**
Pattern 4	**iVerb** of ^∗^ **P1** ^∗^ to ^∗^ **P2**
Pattern 5	**iNoun** of ^∗^ **P1** ^∗^ [by ∣through] ^∗^ **P2**
Pattern 6	**iNoun** of ^∗^ **P1** ^∗^ [with ∣*to*∣on] ^∗^ **P2**
Pattern 7	**iNoun** between ^∗^ **P1** ^∗^ and ^∗^ **P2**
Pattern 8	complex between ^∗^ **P1** ^∗^ and ^∗^ **P2**
Pattern 9	complex of ^∗^ **P1** ^∗^ and ^∗^ **P2**
Pattern 10	**P1** ^∗^ form ^∗^ complex with ^∗^ **iVerb** ^∗^ **P2**
Pattern 11	**P1** ^∗^ **P2** ^∗^ **iNoun**
Pattern 12	**P1** depend of **P2**
Pattern 13	between **P1** and **P2**

We prepared syntax patterns related to PPI based on the syntax patterns proposed by Plake et al. [8]. *P*1 and *P*2 denote the protein names appearing first and later in a sentence, respectively. iNoun and iVerb denote sets of nouns and verbs related to interaction. The number of words substituted by a wildcard ‘ ^∗^’ in a pattern is limited to five. After the training set was divided into subsets based on the existence of *significant keywords* and the structure of the sentence, these syntax patterns were applied to each subset

## Method

**Fig. 2:**
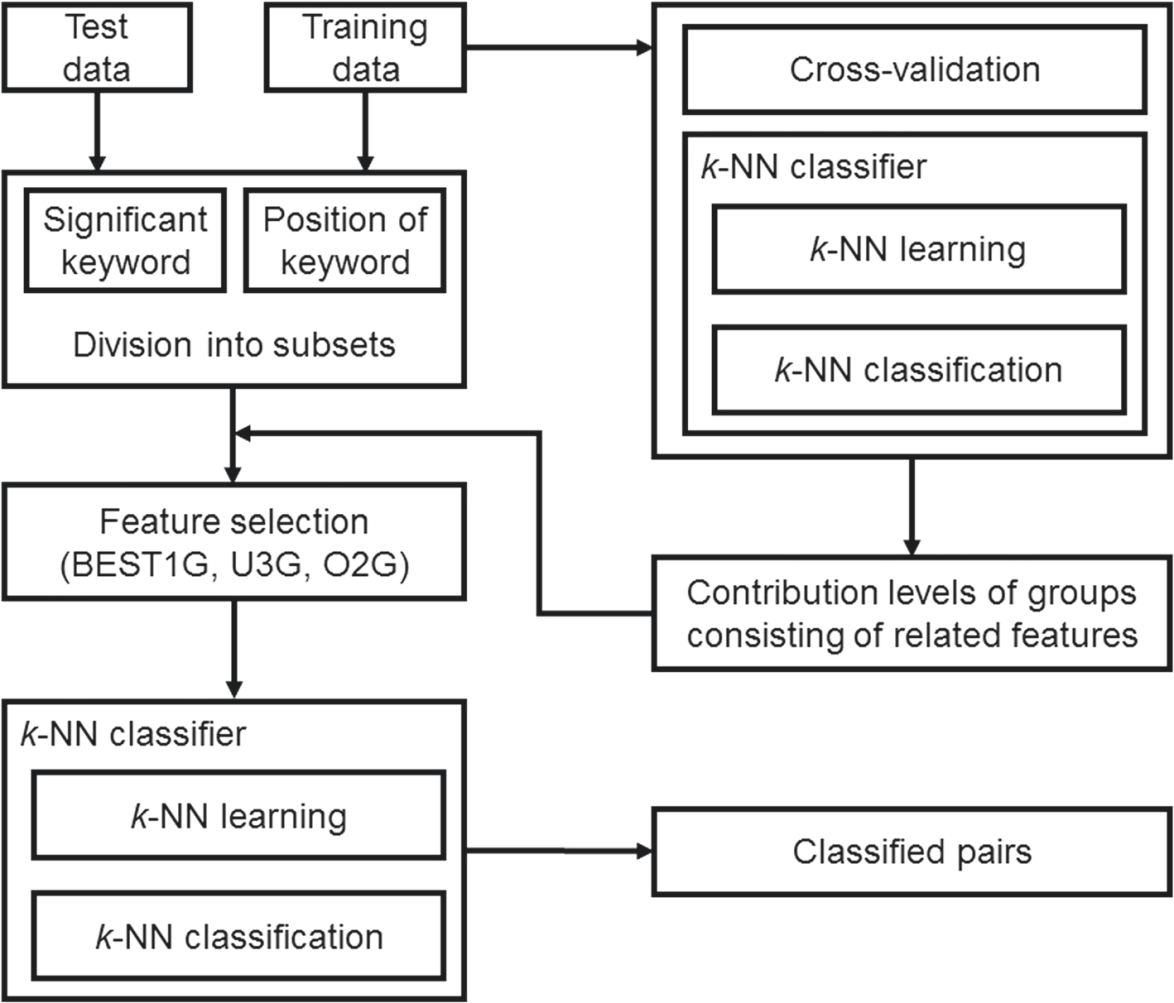
Framework of our PPI extraction system. Our system consists of two phases. First, training set is divided into subsets based on presence of *significant keywords* and the feature *position of keyword*. Second, after cross-validation is performed on the training data to assess the contribution levels of four groups, which consist of related features, feature selection is performed automatically through our three approaches (BEST1G, U3G, O2G). Finally, the *k*-NN classifier is used to classify candidate PPI pairs of test data

Figure 2 shows the framework of our PPI extraction system, which consists of two phases: division of the training set into subsets and PPI prediction based on evaluating the contribution levels (CLs) of the groups consisting of related features. In the first phase, the training data are partitioned into subsets based on the *significant keywords* described below and the feature *position of keyword*. In the second phase, the related features are arranged into groups, and cross-validation is performed on the training data to train the *k*-NN classifier to generate a predictive model, which is utilized to assess the CLs of the groups that consist of related features that indicate the efficiency in the selection of the optimal combination of the features for each group. After the CLs of these groups are estimated, the appropriate features are selected automatically from the following three approaches: focusing on the group with the best CL (BEST1G), unoptimized combination of the three groups with the best CLs (U3G), and optimized combination of the two groups with the best CLs (O2G). Finally, the *k*-NN classifier is used to classify the candidate PPI pairs of the test data.

### Division of the training set into subsets

#### Significant keyword (*SK*)

Since the feature *keyword* (Table 1) performs a noticeable role in identifying whether the sentence contains a PPI, it is utilized in the majority of research related to PPI extraction [9, 10]. Nevertheless, emphasizing only this feature can cause an adverse effect in which other features may not receive appropriate attention or might even be ignored. Consequently, we distinguish between cases when the feature *keyword* contributes significantly to PPI classification and when it does not. We call the *keyword* in the former case *SK*.

Through the observation of the imbalance of the classes of instances when classifying them based on the presence or absence of a certain *keywordK*, we determine whether this feature *keywordK* is *SK* by defining imbalance degree *ID*(*K*) as follows:

1 $$\begin{array}{@{}rcl@{}} ID(K) = N_{P}/N_{N}, \end{array} $$

where *N*_*P*_ and *N*_*N*_ denote the number of positive and negative instances containing *K* in the training set, respectively. *ID*(*K*)=1 means that *K* is completely balanced. On the contrary, *ID*(*K*)=0 (i.e., $\frac {1}{ID(K)}=\infty $) or *ID*(*K*)=*∞* (i.e., $\frac {1}{ID(K)}=0$) means that *K* is completely imbalanced. Thus, when the value of *ID*(*K*) or $\frac {1}{ID(K)}$ is less than predefined threshold *T*, we regard *K* as a *SK*.

#### Position of keyword

In addition to the importance whether a *keyword* is *SK*, since the basic structure of a sentence can be grasped by determining the feature *position of keyword* that signifies the word order of the *keyword* and the pair of protein names in that sentence, this feature should also be stressed. We recognize that if the basic structures of the sentences differ, the features that should be emphasized in PPI classification will also differ. If the feature *position of keyword*’s value is ‘infix’ (i.e., the *keyword* exists between a pair of protein names in the sentence), the sentence structure has the typical Subject-Verb-Object form. For example, in the sentence, “GerE binds to a site on one of these promoters, cotX, that overlaps its -35 region,” (the *keyword* ‘bind’ is the Verb, the protein ‘GerE’ is the Subject, the protein ‘cotX’ is the Object, and the feature *position of keyword*’s value is ‘infix’), since the relation of a protein name and the *keyword* is that of the Subject (or the Object) and the Verb, some features (e.g., *Distance*_*KP*1, *Distance*_*KP*2, and *Distance*_*P*1*P*2 that indicate the distances between protein names and the keyword) perform substantial roles. Conversely, if the feature *position of keyword*’s value is ‘prefix’ or ‘postfix’, the sentence structure is regarded as atypical, such as the parallel expression of protein names, inverted structure, and so forth. For example, in the sentence, “association between cdc25A and cdc2 was detected in the HeLa cells,” (the *keyword* is ‘associate’ obtained by stemming the word ‘association’, the two protein names are ‘cdc25A’ and ‘cdc2’, and the feature *position of keyword*’s value is ‘prefix’), since the relation of a protein name and the *keyword*, which is not that of the Subject (or the Object) and the Verb, is that of inverted structure, some features (e.g, *Distance*_*KP*1, *Distance*_*KP*2, and *Distance*_*P*1*P*2) are not always emphasized. Therefore, the importance of a feature is likely to be varied depending on the structure of the sentence.

#### Division of training set

**Fig. 3:**
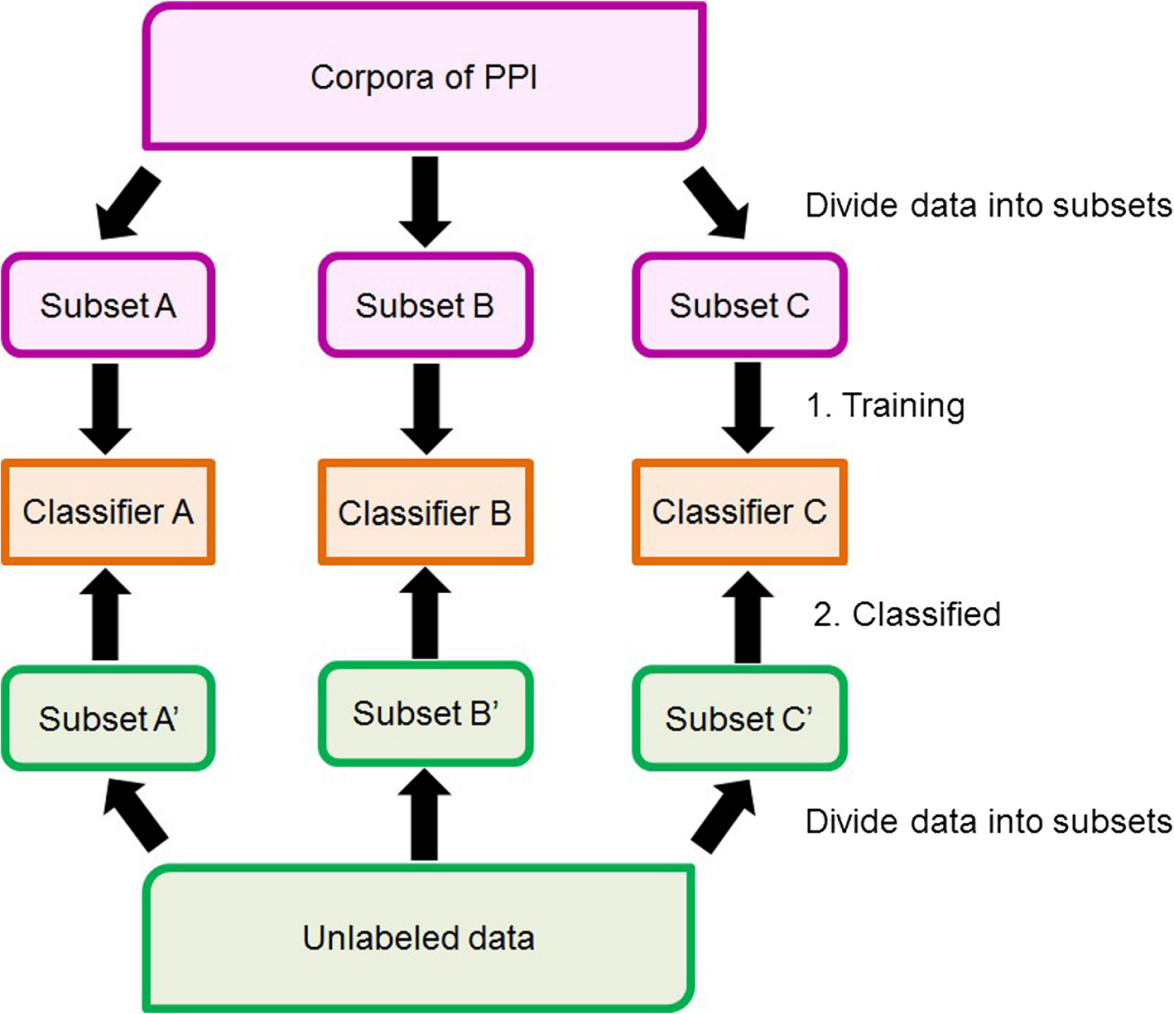
Outline of PPI prediction based on division of training set. Training set was divided into subsets, A, B, and C, based on existence of *significant keyword* and feature *position of keyword*. Three classifiers were generated from every subset. Similarly, unlabeled instances were divided into one of three subsets, A’, B’, and C’, and corresponding classifier was used to identify whether PPIs exist in these instances

Because the features that should be emphasized can vary depending on whether the feature *keyword* is a *SK* and the feature *position of keyword* between the two protein names as stated above, we divide the training set into three subsets, A, B, and C (Table 5), and generate three classifiers from each subset. Similarly, we divide the unlabeled instances into one of three subsets, A’, B’, and C’, and utilize the corresponding classifier to identify whether PPIs exist in these instances. The outline of this process is illustrated in Fig. 3.

**Table 5:** Division of training set

Subset	Significant keyword	Position of keyword
A	Included	Infix
B	Included	Prefix/postfix
C	Not included	Infix/prefix/postfix

The training set was divided into subsets, A, B, and C, based on presence of the *significant keyword* and the feature *position of keyword*

Because the original training set is divided into three subsets, not all the patterns (Table 4) always match each subset. Consequently, we discard the irrelevant and useless patterns beforehand based on the structure of the sentence (Table 6). For subset A, since the feature *position of keyword*’s value is ‘infix’ (i.e., the sentence structure has the typical Subject-Verb-Object form in which the *keyword* that corresponds to a verb exists between two protein names that correspond to a subject and an object), we observed that patterns 7, 8, 9, and 13 (Table 4) do not match this Subject-Verb-Object form. Patterns 7, 8, and 9 stand for phrases *K*- *P*1- *P*2 (the *keyword* is *K* and the protein pair is (*P*1, *P*2)). Similarly, pattern 13 is also unsuitable for subset A. For subset B, because the feature *position of keyword*’s value is ‘prefix’ or ‘postfix’ (i.e., the sentence structure is atypical, such as the parallel expression of protein names, the inverted structure, and so forth), patterns 1, 2, 10, and 12 do not match these sentence structures. As a result, we remove them beforehand for subsets A and B. For subset C, because the *position of keyword*’s value is ‘infix’, ‘prefix’, or ‘postfix’, we do not eliminate any pattern shown in Table 4.

**Table 6:** Removed patterns for each training subset

Subset	Removed patterns
A	Patterns 7,8,9,13
B	Patterns 1,2,10,12
C	No deletions

After the training set was divided into subsets A, B, and C, the syntax patterns (Table 4) we prepared were checked to determine whether they matched each subset. Unsuitable patterns were removed beforehand for subsets A and B. No pattern was excluded for subset C

### Prediction based on evaluating contribution levels of groups consisting of related features

#### Groups consisting of related features

The syntactic features derived from the parsers, features showing the distances between two protein names and the *keyword*, and features showing the positions of two protein names are considered important in PPI classification [4, 6]. However, among some related features (e.g., *Height_P1*, *Height_P2*, and *Height_K* mentioned in Table 3, which depict the heights of the two protein names of the instance and the *keyword* at the constituent parse tree), we cannot affirm intuitively which feature is definitely more important than the others without understanding the data characteristics. Therefore, we arranged the related features into the following four groups to automatically evaluate each one separately in the PPI extraction performance by the CL defined hereinafter:

- Group *G*_1_ consists of three related features: *Distance_KP1*, *Distance_KP2*, and *Distance_P1P2* (Table 2).
- Group *G*_2_ consists of two related features: *Position_P1* and *Position_P2* (Table 2).
- Group *G*_3_ consists of three related features: *Height_P1*, *Height_P2*, and *Height_K* (Table 3).
- Group *G*_4_ consists of three related features: *POS_P1*, *POS_P2*, and *POS_K* (Table 3).

When some features in a group, which consists of related features, are useless and redundant, using all the features in that group can decrease the accuracy of the learning algorithm. In this case, removing some irrelevant features from that group (i.e., feature selection) can improve the PPI extraction performance. Conversely, when all the features in a group are useful, it is unnecessary to remove any feature from that group. By utilizing the original training data, we can determine automatically whether it is necessary to eliminate any feature in any group individually to enhance the PPI extraction performance.

After individually selecting the optimal feature set for each group, we assess the CLs of these groups in the process of training the classifier with the original training data. When some groups greatly contribute to the classifier training, we had better put more focus on these groups. In the opposite case, it is important to give ample consideration to other groups.

#### Cross-validation

**Fig. 4:**
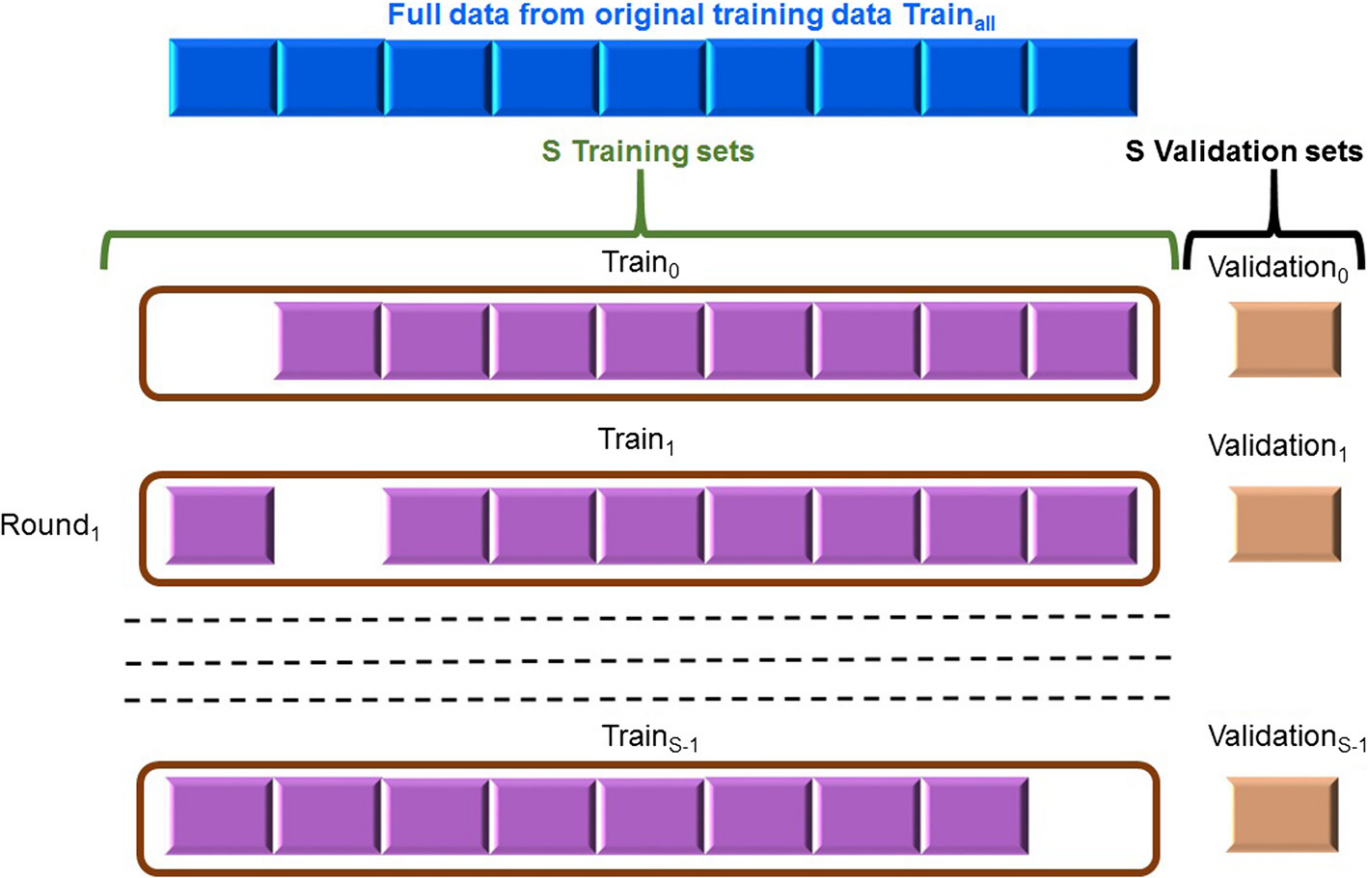
*S*-fold cross-validation (SFCV) performed on original training data. Original training data *T* *r* *a* *i* *n*_all_ was divided into *S* equal-sized partitions *P*_*i*_(*i*=0,⋯,*S*−1) to perform SFCV on it to estimate contribution levels of four groups, *G*_1_, *G*_2_, *G*_3_, and *G*_4_, and perform feature selection

Cross-validation (CV), which is a widespread strategy to estimate the model prediction performance, can also be utilized in feature selection to determine which subsets of features are useful in building good predictive models. In this paper we perform *S*-fold CV (SFCV) on original training data *Train*_all_ to identify the redundant features in groups *G*_1_, *G*_2_, *G*_3_, and *G*_4_, which consist of related features, compute the CLs of these groups, and perform feature selection. In SFCV, original training data *Train*_all_ is divided into *S* equal-sized partitions *P*_*i*_(*i*=0,⋯,*S*−1). In each *Round*_*i*_ of SFCV (*i*=0,…,*S*−1), a combination of *S*−1 partitions is used as training set *Train*_*i*_, defined as *Train*_all_−*P*_*i*_(*i*=0,⋯,*S*−1) (Fig. 4), to train the predictive model that is then validated on the remaining partition, called validation set *Validation*_*i*_(=*P*_*i*_). We discuss it in detail below.

#### Contribution level (CL) of a group consisting of related features

To describe our method more simply from now, we also utilize the abbreviation SFCV, the notations of original training data *Train*_all_, partitions *P*_*i*_, training set *Train*_*i*_, and validation set *Validation*_*i*_(*i*=0,⋯,*S*−1), which were defined in the Cross-validation section.

Because group *G*_1_ contains three related features (*Distance_KP1*, *Distance_KP2*, and *Distance_P1P2*), there are eight ways (2^3^=8) to select features from these features to create eight combinations of features for *G*_1_.

We performed SFCV to train the *k*-NN classifier on original training data *Train*_all_. For each training set *Train*_*i*_(*i*=0,⋯,*S*−1), we trained the *k*-NN classifier with every combination of the features in *G*_1_ (we do not change or remove the features in other groups). Then for each *Train*_*i*_, we search for the optimal combination of features in *G*_1_ that yields the maximum *F*-score on validation set *Validation*_*i*_ (the definition of *F*-score is mentioned in the next section), denoted as *Fcon*_1*i*_ (1 signifies the index of *G*_1_). Similarly, for each *Train*_*i*_, we also look for the optimal combinations of features in groups *G*_2_, *G*_3_, and *G*_4_ separately that yield the maximum *F*-score, denoted as *Fcon*_2*i*_, *Fcon*_3*i*_, and *Fcon*_4*i*_, on *Validation*_*i*_, respectively.

In this paper we define the CL for each group consisting of related features to improve the PPI extraction accuracy. CL is an indicator for determining the efficiency in the selection of the optimal combination of the features for each group. The pseudo-code for the calculation of the CLs of these four groups is shown in Listing ??. *C*_*j*_ denotes the CL of each group *G*_*j*_ (*j*=1,2,3,4). Function *max* returns the maximum value among its arguments. For all values of *i* from 0 to *S*−1, if the maximum among the values of *Fcon*_*ji*_ (*j*=1,2,3,4) is one of group *G*_*t*_, i.e., *Fcon*_*ti*_ (*t*∈{1,2,3,4}), we increase CL *C*_*t*_ of *G*_*t*_.

From the CL values of the four groups output from function *EvalCon*4, if groups *G*_*x*_, *G*_*y*_, and *G*_*z*_ still exist (*x,y*,*z*∈{1,2,3,4}), which have the same CL values, we only recompute the CLs for *G*_*x*_, *G*_*y*_, and *G*_*z*_ by function *EvalCon*3 (Listing ??) without recomputing the CL for the remaining group. After computing the CLs *CL*_*x*_, *CL*_*y*_, and *CL*_*z*_ of *G*_*x*_, *G*_*y*_, and *G*_*z*_ (lines 2–6), which resembles the calculation of the CLs of function *EvalCon*4, we resolve the exception (lines 8–24) in which the CLs of *G*_*x*_, *G*_*y*_, and *G*_*z*_ become equal again. In this exception, we compute *A*_*x*_, *A*_*y*_, and *A*_*z*_ (lines 10–12). *A*_*j*_ (*j*∈{1,2,3,4}) of group *G*_*j*_ denotes the maximum among *Fcon*_*j*0_, *Fcon*_*j*1_,…, and *Fcon*_*j*(*S*−1)_. We regard the CLs of *G*_*x*_, *G*_*y*_, and *G*_*z*_ as identical as *A*_*x*_, *A*_*y*_, and *A*_*z*_, respectively. If all three groups, *G*_*x*_, *G*_*y*_, and *G*_*z*_, still have the same CLs (lines 17–22), we regard the CLs of *G*_*x*_, *G*_*y*_, and *G*_*z*_ as identical as $A^{\prime }_{x}$, $A^{\prime }_{y}$, and $A^{\prime }_{z}$, respectively. In lines 18–20, function *second_max* returns the second maximum among its arguments.

From the CLs of the four groups output from function *EvalCon*4, if only two groups remain with identical CLs, we only recompute the CLs for these groups by function *EvalCon*2 without recomputing the CLs for the remaining groups. Because function *EvalCon*2 resembles function *EvalCon*3, we do not present its code here.





#### Feature selection through three approaches (BEST1G, U3G, O2G)

The ultimate goal of automatic PPI extraction from articles is extracting PPIs from any new unseen text with high predictive accuracy. In addition to PPI extraction accuracy, reducing the computational time for training and testing a PPI extraction system is also crucial. Tikk et al. [11] compared the performances of diverse kinds of kernels on a large-scale database called Medline, which contains nearly 120-M sentences. They reported that when the top three kernels (all path graph kernel, shallow linguistic kernel, and *k*-band shortest path spectrum kernel) that show the best performance were applied to Medline on a single processor, their runtimes were about 45, 141, and 4 days, respectively. Including the time to parse sentences, their runtimes changed to 226, 147, and 185 days, respectively. In other words, to extract PPI on Medline, we need about half a year. Consequently, lowering the computational time becomes critical for PPI extraction tasks. Landeghem et al. [2] also argued that we have to consider a trade-off between PPI extraction accuracy and computational time to decrease the amount of computational resources utilized by machine learning.

Based on the CL values of four groups, *G*_1_, *G*_2_, *G*_3_, and *G*_4_, we employ the following three approaches: focusing on the group with the best contribution level (BEST1G), unoptimized combination of three groups with the best contribution levels (U3G), and optimized combination of two groups with the best contribution levels (O2G) to automatically perform feature selection to enhance PPI extraction accuracy. For the above reason, we also take into account reducing the computational time by feature selection. We briefly describe the advantages of our three approaches below.

**BEST1G:** We only performed feature selection automatically on the group with the best CL among the four groups. Although we also aim at improving PPI extraction accuracy, our concern with this approach is mainly about limiting the computational time by feature selection as much as possible. Therefore, compared with U3G and O2G, the advantage of BEST1G is the least computational time due to feature selection. However, since BEST1G is not guaranteed to always attain better extraction accuracy than U3G and O2G, it is suitable for a large-sized dataset in which effectively decreasing the computational time is always the most required factor.

**U3G:** We merged all the features in the three groups with the best CLs among the four groups. Then we automatically performed feature selection on these merged features from these three groups by a greedy algorithm. Although this greedy strategy generally does not produce an optimal subset of these features, it may yield a locally optimal subset of them. We want to exploit the information from the features in these three groups to improve the extraction accuracy without wasting time performing feature selection by exhaustively searching for all their combinations. Hence, compared with BEST1G and O2G, the advantage of U3G is maintaining a trade-off between extraction accuracy and computational time due to feature selection. However, U3G is also not guaranteed to always achieve better extraction accuracy than BEST1G and O2G. As a result, U3G is suitable for a medium-sized dataset in which balancing extraction accuracy and computational time is necessary.

**O2G:** We merged all the features in the two groups with the best CLs among the four groups. Then we automatically performed feature selection on these merged features from these two groups by exhaustively searching for all the combinations of these features. Consequently, we assume that O2G performs better with respect to extraction accuracy than BEST1G and U3G. The disadvantage of O2G is higher computational times due to feature selection than BEST1G and U3G when applying O2G, BEST1G, and U3G to a medium-sized dataset or a large-sized dataset. As a result, O2G is suitable for a small-sized dataset in which effectively increasing the extraction accuracy is always the most required factor.

We describe the implementation details of BEST1G, U3G, and O2G below.

##### BEST1G

First, we selected group *G*_*j*_ having the best CL among the four groups. For all the values of *i* from 0 to *S*−1, we then searched for the maximum of *Fcon*_*ji*_ and its corresponding combination of features in *G*_*j*_ when applying SFCV and *k*-NN to training set *Train*_*i*_.

##### U3G

First, we selected three groups having the best CLs among the four groups and merged all the features in them. Assume that the order of CLs *C*_*a*_, *C*_*b*_, and *C*_*c*_ of the three corresponding selected groups *G*_*a*_, *G*_*b*_, and *G*_*c*_ is *C*_*a*_>*C*_*b*_>*C*_*c*_. For each training set *Train*_*i*_(*i*=0,⋯,*S*−1), we performed *k*-NN by gradually removing the features in these three groups in the order of their CLs (i.e., we eliminated the features in *G*_*a*_ first, then those in *G*_*b*_, and finally those in *G*_*c*_), and if the *F*-score, denoted as *F*_*i*_, on validation set *Validation*_*i*_ improved even slightly, we conclude immediately that this improved value of *F*_*i*_, denoted as *Fu*_*i*_, is the unoptimized value for *Train*_*i*_.

Next, for all the values of *i* from 0 to *S*−1, we searched for the maximum of *Fu*_*i*_ and its corresponding combination of features in these three groups.

##### O2G

First, we selected two groups having the best CLs among the four groups and merged all the features in them. For each training set *Train*_*i*_(*i*=0,⋯,*S*−1), we performed *k*-NN with every combination of the features in these two merged groups. Then, for each training set *Train*_*i*_, we searched for the optimal combination of the features in these two merged groups that yields the maximum *F*-score, denoted as *Fo*_*i*_, on validation set *Validation*_*i*_.

Next, for all the values of *i* from 0 to *S*−1, we searched for the maximum of *Fo*_*i*_ and its corresponding combination of features in these two merged groups.

## Results and Discussion

### Datasets

We utilized all the datasets from four typical PPI-annotated corpora: LLL [12], HPRD50 [13], IEPA [14], and AIMed [15] (except BioInfer [16]). Table 7 shows the number of positive and negative instances in them. In addition to the 200 PubMed abstracts in AIMed that were manually annotated for interactions between human genes and proteins, 30 other abstracts without PPIs were added to AIMed as negative instances. HPRD50 is comprised of 50 abstracts, in which the human gene and protein names were automatically identified by ProMiner software. IEPA was created from 303 PubMed abstracts, each of which contains a specific pair of co-occurring chemicals. The LLL corpus contains 77 sentences and was the shared dataset for the Learning Language in Logic 2005 challenge (LLL05). The LLL domain is the gene interactions of Bacillus subtilis. These corpora carry information about named entities from biological domains and annotated PPIs. Nevertheless, they differ from one another. For example, with respect to the scope of the annotated entities, most of them typically contain proteins and genes, some contain RNAs, but IEPA contains only chemicals. The policies of entity annotation and interaction annotation among these copora are also slightly different [17]. Pyysalo et al. [17] reported that although the entity annotation of the types relevant to the corpus is exhaustive only in AIMed and BioInfer, entity annotation is merely based on lists of entity names or the named entity recognizer output in other corpora. They also indicated that the differences in interaction annotation are even greater than those in entity annotation, e.g., only BioInfer and IEPA contain information identifying the words that state an interaction, and all but HPRD50 specify the direction of the interactions. They converted these corpora into a unified XML format, which we utilized in our study, with a very simple structure to make the corpora easily accessible to users. In the unified XML format, each corpus is comprised of documents that are abstracts of articles, each document is comprised of sentences, each sentence might contain some proteins, and the names and relations of proteins are annotated by some attributes of this XML format.

**Table 7:** Number of positive and negative instances in four corpora: LLL, HPRD50, IEPA, and AIMed

Corpus	LLL	HPRD50	IEPA	AIMed
Positive instances	164	163	335	1000
Negative instances	166	270	482	4834

Four corpora, LLL, HPRD50, IEPA, and AIMed, were converted into a unified XML format with a very simple structure by Pyysalo et al. [17] to make the corpora easily accessible to users. Number of positive instances (interacting protein pairs) and negative instances (non-interacting protein pairs) in each corpus is shown

We regard the PPI extraction task as a binary classification in which interacting protein pairs are considered positive instances and vice versa. If a sentence contains *n* proteins, ${n \choose 2}$ instances (i.e., protein pairs) are generated. In this paper, the two protein names of a candidate PPI instance and the other proteins in the same sentence are renamed as *P*1, *P*2, and *P*0 to blind the learner to allow direct comparison to earlier studies. By utilizing the information contained in the attributes of the unified XML format of these corpora, we parsed all the sentences in them to extract the features.

BioInfer has an especially extensive annotation policy that combines three types of annotations: termed entity, entity relationship, and dependency. Some sentences of this corpus were annotated with protein names that do not contain contiguous tokens. For instance, in the phrase, “When liver- or islet-type glucokinase was transiently expressed in COS-7 cells” from sentence BioInfer.d43.s3, two protein names are annotated, “liver-type glucokinase” and “islet-type glucokinase,” and “liver-type glucokinase” is annotated as a protein reference despite its discontinuous name. In this paper, the two protein names of a candidate PPI instance and the other proteins in the same sentence are assumed to contain only contiguous tokens. Although we currently cannot tackle these non-contiguous protein names of the BioInfer corpus, which is why we do not analyze the dataset of this corpus, we intend to deal with this problem in the future.

### Evaluation methods

To allow for direct comparison to earlier studies, we evaluated the performance of our method by 10-fold document-level cross-validation (10FDLCV). In each round of 10FDLCV, one of the ten partitions containing 10 % of the documents is used as a test set, and the combination of the remaining nine partitions containing 90 % of the documents is used as a training set. SFCV, which is described in the Method section, is performed as 9FDLCV (*S* is set to 9). We also adopted the One Answer per Occurrence strategy in which a correct interaction has to be extracted for each occurrence of the instance. The threshold *T*’s value (the Method section) is set to 0.18.

For classifying PPI, we use the *k*-NN algorithm mentioned above by normalizing the value of each feature before computing the Euclidean distance between two feature vectors. The values of the features are normalized so that they all lie between 0 and 1 and the features on different scales have the same impact on the distance function. If two categorical features are identical (or different), we consider the difference between them as 0 (or 1).

For small-sized corpora, LLL, HPRD50, and IEPA, based on the rule of thumb in machine learning that chooses parameter *k* of *k*-NN to be near the square root of the size of the training set, we chose *k* to be odd and equal to 19, 21, and 29 (in binary classification, we choose an odd *k* of *k*-NN to avoid ties). For a larger AIMed corpus containing the most highly imbalanced PPI data, if the rule of thumb is applied, *k* is too big, about 71–73, and the computation cost (i.e., the computation of the distances among instances) also increases greatly with this value of *k*. Therefore, for the AIMed corpus, we performed cross-validation on the training data with a range of value of *k*, and chose *k* that equals 11 based on the lowest root mean square error.

Precision (P), recall (R), and harmonic value *F*-score (F) are used as evaluation measures, which are defined as follows:

2 $$\begin{array}{@{}rcl@{}} P \ &=& TP/(TP+FP) \end{array} $$

3 $$\begin{array}{@{}rcl@{}} R \ &=& TP/(TP+FN) \end{array} $$

4 $$\begin{array}{@{}rcl@{}} F \ &=& 2*P*R/(P+R), \end{array} $$

*TP*, *FP*, and *FN* denote the number of true positives, false positives, and false negatives, respectively. Precision is the percentage of correct predictions from all the instances predicted as positive. Recall is the percentage of correctly predicted positive instances from all positive instances.

### Experiment results

Table 8 shows the results of our three approaches (BEST1G, U3G, O2G). As a baseline, we also added the results when only *k*-NN was applied, and feature selection using the CLs of the groups consisting of related features was not performed. Our three approaches considerably improved all the *F*-score results on all the corpora compared with the case that only uses *k*-NN without performing feature selection that utilizes the CLs of these groups.

**Table 8:** Experiment results of our three approaches: BEST1G, U3G, O2G

Corpus	LLL			HPRD50			IEPA			AIMed		
(%)	P	R	F	P	R	F	P	R	F	P	R	F
*k*-NN	71.6	79.8	74.4	71.9	62.4	65.9	**68.4**	66.6	67.2	**52.1**	35.9	42.3
BEST1G	**74.7**	**82.2**	**76.5**	72.5	72.6	71.6	67.7	71.3	69.2	50.8	**40.9**	**45.1**
U3G	74.6	80.7	75.9	72.5	72.6	71.6	68.3	**71.7**	**69.8**	49.5	40.5	44.4
O2G	**74.7**	**82.2**	**76.5**	**73.0**	**74.3**	**72.6**	68.1	71.3	69.5	50.2	39.8	44.3

Precision (P), recall (R), *F*-score (F) results of our three approaches (BEST1G, U3G, O2G) evaluated by 10-fold document-level cross-validation on four corpora, LLL, HPRD50, IEPA, and AIMed, shown in the second, third, and fourth row. As a baseline, in the first row, we add results when only *k*-NN is applied, and feature selection using contribution levels of groups consisting of related features was not performed. Precision (P), recall (R), and *F*-score (F) values are shown by percentage (%). Bold typeface shows best results per corpus in terms of precision, recall, and *F*-score

Compared with BEST1G, although U3G attains a better result on IEPA or an equivalent result on HPRD50, BEST1G outperforms U3G on LLL and AIMed in terms of *F*-score. BEST1G learned all the possible combinations of the features in the group with the best CL, whereas there are cases where U3G can immediately halt the feature selection completely when only a feature in the group with the best CL is removed and the *F*-score on the validation set is only slightly improved. Therefore, the BEST1G results are generally better than those of U3G.

Conversely, since O2G exhaustively searches for all the combinations of the features in the two merged groups having the best CLs, O2G exceeds BEST1G on HPRD50 and IEPA and greatly surpasses U3G on LLL and HPRD50. Generally, despite the highest computational time due to feature selection when applied to the medium-sized IEPA corpus or the large-sized AIMed corpus, O2G performs the best among our three approaches, as we assumed in the Method section.

However, depending on the characteristics of the corpus, the best performance belongs to BEST1G, U3G, and O2G on AIMed, IEPA, and HPRD50, respectively, in terms of *F*-score. Both BEST1G and O2G performed best on LLL. O2G failed to attain the best *F*-scores on IEPA and AIMed, as we assumed in the Method section, because the heterogeneity among corpora can lead to heterogeneous evaluation results. For example, unlike other corpora, 30 abstracts without PPIs were intentionally added to AIMed as negative instances. As a result, the percentage of sentences that have no entities is 18 % in AIMed, but it is 0 % in the other corpora. The percentage of sentences that have no interactions is 69 % in AIMed, but it is less than or equal to 38 % in the other corpora. The ratio between the number of positive instances and all the instances in the highly imbalanced AIMed is too low, about only 17.1 % compared with the other corpora. Additionally, unlike other corpora, the scope of the annotated entities in IEPA is chemicals. Therefore, due to the enormous differences among the corpora, in a few cases, O2G may not be the best approach, even though overall it is superior to BEST1G and U3G with respect to extraction accuracy.

Moreover, the *F*-score results of O2G on IEPA and AIMed still surpass those when only *k*-NN is applied. As described in the Method section, with a large-sized corpus like AIMed in which effectively decreasing the computational time is always the most required factor, we should apply BEST1G because it has the lowest computational time. With a medium-sized corpus like IEPA in which we need a trade-off between extraction accuracy and computational time, we should utilize U3G. With small-sized copora like LLL and HPRD50 in which effectively increasing extraction accuracy is always the most necessary factor, O2G remains the best choice.

### Comparison with other related research

The performance comparison of our three approaches (BEST1G, U3G, O2G) with other related research is shown in Table 9. The results of the co-occurrence and rule-based methods are also listed in Table 9 as a baseline. Fundel et al., who proposed the RelEx system [13], applied a small number of simple rules to dependency parse trees to extract PPI. Kabiljo et al., who proposed the AkanePPI(B) system [18], utilized support vector machines with tree kernels to extract rules for PPI extraction by a combination of deep syntactic parser Enju and a shallow dependency parser. Our approaches (BEST1G, U3G, O2G) are mostly far better than these co-occurrence and rule-based methods on all the corpora in terms of *F*-score.

**Table 9:** Comparison of our three approaches (BEST1G, U3G, O2G) with other systems

Corpus		LLL			HPRD50			IEPA			AIMed		
(%)		P	R	F	P	R	F	P	R	F	P	R	F
	BEST1G	**74.7**	**82.2**	76.5	72.5	72.6	71.6	67.7	71.3	69.2	50.8	40.9	45.1
	U3G	74.6	80.7	75.9	72.5	72.6	71.6	**68.3**	**71.7**	**69.8**	49.5	40.5	44.4
Feature-	O2G	**74.7**	**82.2**	76.5	**73.0**	**74.3**	**72.6**	68.1	71.3	69.5	50.2	39.8	44.3
based	Landeghem et al. [2]	72.0	73.0	73.0	60.0	51.0	55.0	64.0	70.0	67.0	49.0	44.0	46.0
methods	Liu et al. [1]			**78.1**			64.9			62.1	**63.4**	**48.8**	**54.7**
	Yakushiji et al. [19]										33.7	33.1	33.4
Kernel-	Airola et al. [3]	72.5	87.2	76.8	64.3	65.8	63.4	**69.6**	82.7	**75.1**	52.9	61.8	56.4
based	Miwa et al. [4]	**77.6**	86.0	80.1	**68.5**	76.1	70.9	67.5	78.6	71.7	55.0	**68.8**	**60.8**
methods	Tikk et al. [11]	69.3	**93.2**	78.1	62.2	**87.1**	**71.0**	58.8	**89.7**	70.5	50.1	41.4	44.6
	Qian et al. [5]			**84.6**			68.8			69.8	**59.1**	57.6	58.1
Co-occurrence	Airola et al. [3]	55.9	100.0	70.3	38.9	100.0	55.4	40.8	100.0	57.6	17.8	100.0	30.1
Rule-based	RelEx [13, 17]	82.0	72.0	77.0	76.0	64.0	69.0	74.0	61.0	67.0	40.0	50.0	44.0
methods	Kabiljo et al. [18]	76.7	40.2	52.8	52.0	55.8	53.8	66.2	51.3	57.8	29.1	52.9	37.5

Performance comparison of our three approaches (BEST1G, U3G, O2G) with other related research on four corpora: LLL, HPRD50, IEPA, and AIMed. Co-occurrence and rule-based methods results are also listed as a baseline. Precision (P), recall (R), and *F*-score (F) values are shown by percentage (%). Bold typeface shows best results of feature-based and kernel-based methods per corpus in terms of precision, recall, and *F*-score

Compared with the feature-based methods, BEST1G, U3G, and O2G outperformed their performances on LLL, HPRD50, and IEPA (except compared with *F*-score of the method by Liu et al. [1] on LLL).

Compared with the kernel-based methods, BEST1G, U3G, and O2G achieved *F*-score results on par with two of these methods (Airola et al. [3], Tikk et al. [11]) on LLL and with three of these methods (Miwa et al. [4], Qian et al. [5], Tikk et al.) on IEPA. In terms of precision, our three approaches outperformed two of the kernel-based methods (Airola et al., Tikk et al.) on LLL and two of the kernel-based methods (Miwa et al., Tikk et al.) on IEPA. Each of the methods has its own disadvantages and advantages. Therefore, no method is almighty and powerful for all the corpora. For each kernel-method, there exists a corpus for which that particular method is the best compared with other methods (e.g., the methods proposed by Airola et al., Miwa et al., and Qian et al. outperformed other methods in terms of *F*-score on IEPA, AIMed, and LLL, respectively), and there exist corpora for which that particular method is not suitable (i.e., the *F*-score results of that particular method on these corpora are not good). In the method by Tikk et al., there is no corpus showing remarkably high result of *F*-score compared with our approaches and other methods.

Especially in case of the HPRD50 corpus, our approaches greatly surpassed the feature-based methods as well as the kernel-based ones. In terms of *F*-scores on HPRD50, O2G exceeds the best results of the feature-based methods and the kernel-based ones (i.e., 64.9 % and 71.0 %) by 7.7 % and 1.6 %, respectively. In that respect, our approaches have a distinct unique value compared with other related research.

Conversely, in case of the AIMed corpus, although our approaches performed fairly better than the Tikk et al. and Yakushiji et al. [19] methods or on par with the method by Landeghem et al. [2], our approaches are not better than other feature-based and kernel-based methods in terms of *F*-scores. However, a direct comparison among different systems on AIMed is not straightforward due to the difference in data preprocessing, remarkably different interpretations related to the number of interacting or non-interacting pairs in the AIMed corpus [3], learning methods, parameter tuning, and different evaluation methods [11]. For instance, Liu et al. [1] identified two more interacting and 40 fewer non-interacting protein pairs than our study in AIMed. Similarly, 164 fewer non-interacting protein pairs were reported in the work of Landeghem et al. Moreover, since the self-interactions (59 instances) of the AIMed corpus are not regarded as PPI candidates and were removed from the corpus prior to evaluation in the methods proposed by Liu et al., Airola et al., Miwa et al., and Qian et al., the *F*-score results of these systems would be higher than our method in which we did not discard self-interactions in the preprocessing step. These differences could boost the performance of these studies. Further, we did not utilize dependency information, like the methods proposed by Liu et al., Landeghem et al., Airola et al., Miwa et al., Qian et al., and Tikk et al., or the deep syntactic information (called predicate argument structure), like the method proposed by Miwa et al., which are derived from a dependency parser or a deep parser, respectively, and can increase the accuracy of predictive models [4].

## Conclusion

In this paper we propose a novel method for automatic PPI extraction from research articles. Our method automatically implements feature selection based on evaluating the CLs of the groups that consist of related features to enhance the extraction performance. Our three approaches (BEST1G, U3G, O2G) attain comparable performance in all the corpora and achieve better results in the HPRD50 corpus than the other previous research. In addition, our three approaches always gain better *F*-scores in all the corpora than when only using *k*-NN without utilizing the CLs of the groups that consist of related features.

In realistic PPI datasets, there are far fewer interacting protein pairs than non-interacting ones. The imbalance of the PPI data can compromise the process of learning. Dealing with the imbalance of PPI data is highly challenging [20]. For future work, we plan to design a more efficient method to resolve this problem. Moreover, we intend to design an ensemble that is comprised of dissimilar kernels, devise more effective representations of instances, and explore novel useful features to improve the PPI extraction performance. We also plan to create a predictive model for PPI extraction based on deep learning techniques by combining with kernels to extract PPIs from such large text collections as Medline.
